# Detection of Structural Variants in Circulating Cell-Free DNA from Sarcoma Patients Using Next Generation Sequencing

**DOI:** 10.3390/cancers12123627

**Published:** 2020-12-03

**Authors:** Lauren Mc Connell, Jana Gazdova, Katja Beck, Shambhavi Srivastava, Louise Harewood, JP Stewart, Daniel Hübschmann, Albrecht Stenzinger, Hanno Glimm, Christoph E. Heilig, Stefan Fröhling, David Gonzalez

**Affiliations:** 1 Patrick G Johnston Centre for Cancer Research, Queen’s University, Belfast BT9 7AE, UK; lmcconnell13@qub.ac.uk (L.M.C.); j.gazdova@qub.ac.uk (J.G.); S.Srivastava@qub.ac.uk (S.S.); L.Harewood@qub.ac.uk (L.H.); p.stewart@qub.ac.uk (J.S.); 2 Department of Translational Medical Oncology, National Center for Tumor Diseases (NCT), 69120 Heidelberg, Germany; katja.beck@nct-heidelberg.de (K.B.); christoph.heilig@nct-heidelberg.de (C.E.H.); stefan.froehling@nct-heidelberg.de (S.F.); 3 German Cancer Research Center, 69120 Heidelberg, Germany; albrecht.stenzinger@med.uni-heidelberg.de; 4 Computational Oncology, Molecular Diagnostics Program, National Center for Tumor Diseases (NCT) Heidelberg and DKFZ, 69120 Heidelberg, Germany; d.huebschmann@dkfz-heidelberg.de; 5 Heidelberg Institute for Stem Cell Technology and Experimental Medicine (HI-STEM), 69120 Heidelberg, Germany; 6 Institute of Pathology, University Hospital Heidelberg Im Neuenheimer Feld 224, 69120 Heidelberg, Germany; 7 Department of Translational Medical Oncology, National Center for Tumor Diseases (NCT) Dresden and German Cancer Research Center (DKFZ), 01307 Dresden, Germany; hanno.glimm@nct-dresden.de; 8 Center for Personalized Oncology, National Center for Tumour Diseases (NCT) Dresden and University Hospital Carl Gustav Carus Dresden at TU Dresden, 01307 Dresden, Germany; 9 Translational Functional Cancer Genomics, National Center for Tumor Diseases (NCT) and German Cancer Research Center (DKFZ), 69120 Heidelberg, Germany; 10 German Cancer Consortium (DKTK), 01307 Dresden, Germany; 11 Belfast Health & Social Care Trust, Belfast BT9 7AB, UK

**Keywords:** next generation sequencing, sarcoma, cell-free DNA, translocations

## Abstract

**Simple Summary:**

Accurate diagnosis of sarcoma can be difficult as there are over 100 different subtypes. Approximately one third of sarcomas are characterised by subtype-specific genetic variants, which are routinely detected by the molecular testing of tumour biopsies. Recent studies have shown the potential use of next generation sequencing (NGS) for variant detection in circulating tumour DNA (ctDNA), which is DNA released from tumour cells into the bloodstream. Our feasibility study is the first to demonstrate the application of a custom NGS gene panel, targeting genetic variants in several sarcoma subtypes using ctDNA samples.

**Abstract:**

Circulating tumour DNA (ctDNA) analysis using next generation sequencing (NGS) is being implemented in clinical practice for treatment stratification and disease monitoring. However, using ctDNA to detect structural variants, a common occurrence in sarcoma, can be challenging. Here, we use a sarcoma-specific targeted NGS panel to identify translocations and copy number variants in a cohort of 12 tissue specimens and matched circulating cell-free DNA (cfDNA) from soft tissue sarcoma patients, including alveolar rhabdomyosarcoma (*n* = 2), Ewing’s Sarcoma (*n* = 2), synovial sarcoma (*n* = 2), extraskeletal myxoid chondrosarcoma (*n* = 1), clear cell sarcoma (*n* = 1), undifferentiated round cell sarcoma (*n* = 1), myxoid liposarcoma (*n* = 1), alveolar soft part cell sarcoma (*n* = 1) and dedifferentiated liposarcoma (*n* = 1). Structural variants were detected in 11/12 (91.6%) and 6/12 (50%) of tissue and plasma samples, respectively. Structural variants were detected in cfDNA at variant allele frequencies >0.2% with an average sequencing depth of 1026×. The results from this cohort show clinical potential for using NGS in ctDNA to aid in the diagnosis and clinical monitoring of sarcomas and warrant additional studies in larger cohorts.

## 1. Introduction

Sarcomas are a rare group of heterogeneous tumours that arise within bone or soft tissues and account for approximately 1% of all adult cancers and 20% of paediatric solid malignancies [1,2,3]. Soft tissue sarcomas (STS) are more common than bone, constituting approximately 90% of all diagnosed sarcoma cases and can be broadly divided into two categories: those with specific genetic alterations and those displaying multiple complex karyotypic abnormalities [1,4,5]. Most sarcoma-related genetic alterations represent chromosomal translocations that result in fusion genes and it has been estimated that around one third of sarcomas carry a detectable driver fusion gene [6,7,8].

Some sarcoma subtypes display recurrent, often pathognomonic fusion genes which when identified, can support the diagnosis of specific sarcomas [9,10]. Additionally, copy number changes in particular genes can support differential diagnosis [11]. Some fusion genes and copy number alterations can also serve as therapeutic targets, therefore making the identification of these structural variants in sarcoma highly important, for both diagnosis and treatment options [12].

Material for diagnostic examination of solid tumours is routinely obtained through fine-needle biopsies or tissue resections that are subsequently formalin-fixed and paraffin-embedded (FFPE). Conventional haematoxylin and eosin (H&E) staining and immunohistochemistry (IHC), molecular analysis using reverse-transcription polymerase chain reaction (RT-PCR), fluorescent in situ hybridisation (FISH) and more recently, next-generation sequencing (NGS) are all valuable diagnostic tools [13,14]. NGS is a high-throughput, cost-effective tool allowing for multiple chromosomal regions to be sequenced in parallel to interrogate a wide range of genetic variations including single nucleotide variants (SNVs), insertion–deletions (indels), translocations and copy number variants (CNV) [15,16].

Whilst whole genome and exome sequencing allow for the discovery of novel genomic alterations, targeted NGS is a cost-effective technology for detection of known structural variants, allowing for specific regions of interest to be enriched and sequenced at deeper coverage, making it more suitable for use in scanty specimens such as FFPE biopsies and circulating cell-free DNA (cfDNA) [17].

Studies in various cancer types, have demonstrated the potential utility of identifying and monitoring tumour-specific mutations in cfDNA isolated from plasma. Successful utilisation of circulating tumour DNA (ctDNA) for disease prognostication and association with response to therapy, has mainly relied on the identification of highly recurrent SNVs [18]. Sarcomas however are less amenable to such approaches as they harbour complex genomic alterations, such as translocations and copy number changes, and generally have a low mutational burden [19,20]. Furthermore, many of the current approaches for ctDNA analysis require prior knowledge of the tumour genotype in order to design highly sensitive tumour-specific assays, and therefore are not suitable for differential diagnostic purposes.

Recent investigations into the use of ctDNA in sarcomas with translocations are limited but have shown promise. Shukla et al. and Shulman et al. have both investigated *EWSR1* translocation detection in ctDNA using targeted NGS [21,22]. Shukla et al. used DNA probes targeting all coding exons of *STAG2* and *TP53* and *EWSR1* intron 7–13, whereas Shulman et al. used a custom panel targeting *EWSR1*, *FUS*, *CIC*, *CCNB3* and the coding regions of *TP53* and *STAG2* [21,22].

For this study, we used a pan-sarcoma fusion gene capture-based NGS panel, designed for use with genomic DNA and previously validated with FFPE samples, on a cohort of sarcomas with matched tumour tissue and plasma samples [23]. To the best of our knowledge, this is the first investigation using a validated pan-sarcoma gene panel targeting 87 fusion genes and 7 sarcoma-related CNVs, to detect structural variants from cfDNA. Our aims were to assess the feasibility of a comprehensive sarcoma capture-based NGS assay to detect clinically relevant fusion genes and copy number variations in ctDNA samples.

## 2. Results

### 2.1. Patient Information

Twelve patients with metastatic sarcoma with a wide range of structural variants were selected for sampling (Table 1). All patients had a reported structural variant, measurable metastatic disease at ctDNA sampling and were receiving treatment prior to sampling. The average DNA yield from fresh frozen tissue and plasma, following extraction, was 115 ng (range 30–158 ng) and 7.9 ng (range 3.2–14.4 ng), respectively.

### 2.2. Matched Tumour Tissue Sequencing Metrics

The 12 matched tumour samples (fresh frozen) produced an average of 12,220,362 unique reads passing the filter with a mean target coverage depth of 2390× (range 675×–4374×). The sarcoma cfDNA samples produced an average of 12,677,104 unique reads passing the filter and a mean target coverage depth of 1026× (range 842×–1270×). A summary of sequencing metrics for both sequencing runs can be found in Table 2.

### 2.3. cfDNA and Matched Tumour Sample Variant Analysis

Ten translocations and one *MDM2* amplification out of 12 structural variants (92% detection rate) were detected from the matched tumour samples. Read depth for these samples ranged from 675× to 4374×.

**Figure 1:**
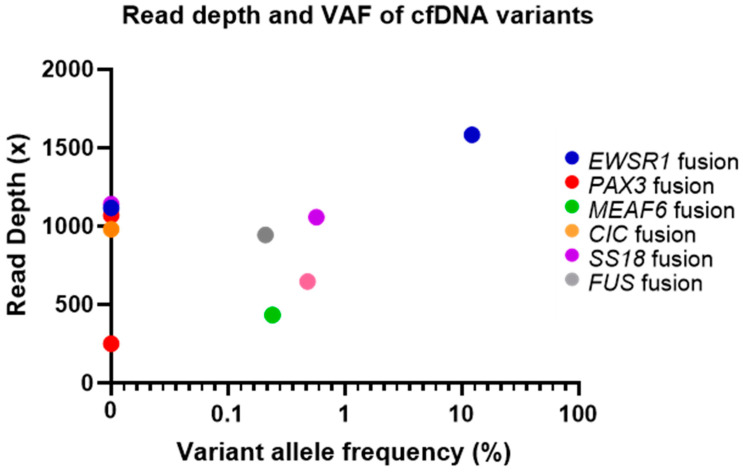
Fusion genes detected and estimated VAF (%) for each circulating tumour DNA (ctDNA) sample. Note: VAF = variant allele frequency.

Out of the 12 sarcoma cfDNA samples, five sarcoma fusion genes (*EWSR1-ATF1*, *MEAF6-PHF1*, *PAX3-FOXO1*, *EWSR1-FLI1*, *SS18-SSX2*) and one *MDM2* amplification were detected following analysis (50% of structural variants detected). Variant allele frequency (VAF), calculated as described in the methods section, ranged from 0.21 to 13.83%. The sarcoma fusion genes detected and the estimated VAF for each cfDNA sample can be seen in Figure 1.

Structural variants were not detected in either cfDNA or matched tumour tissue samples in one case (M5), as the precise fusion breakpoint was not targeted by the gene panel. No additional SNV mutations were detected in either tumour tissue samples or the matched cfDNA samples. Additionally, no translocations or copy number variations were reported in any ctDNA sample that did not correspond with matched tissue, similar to results reported previously using this gene panel in FFPE tissue samples [23], resulting in a specificity of 100%.

## 3. Discussion

A total of 12 matched tumour samples were sequenced and 11 structural variants were detected (91.6%) in the fresh frozen material, whereas six variants out of those 11 (55%) were detected from the matched ctDNA. The average unique sequencing depth for the tissue and cfDNA samples was 2390× and 1026×, respectively. Five translocations out of the 10 fusion-positive samples confirmed by tissue sequencing were detected in the corresponding cfDNA, as well as *MDM2* amplification in a case of *MDM2*-amplified liposarcoma. Translocations were detected from ctDNA at a VAF ranging from 0.21 to 13.83%. Previous work performed by Shulman et al. achieved an average unique sequencing depth of 579× (range 151.8–1311.2×) and identified *EWSR1* translocations in 41 out of 77 patients (53.3%) with ctDNA levels ranging from 1.4 to 43.2% [22]. Shukla et al. achieved much deeper coverage with an average unique sequencing depth of 3754x and identified 14 out of 17 *EWSR1* translocations (82.3%) in patients with ctDNA levels ranging from 0.16 to 15.67% [21]. In the latter study, the authors used a starting cfDNA input ranging from 15 to 421 ng whereas cfDNA input for our current study ranged from 3.2 to 14.4 ng [21]. These data suggest that increasing the initial amount of cfDNA and the corresponding increase in sequencing depth, can lead to additional translocations being detected in cfDNA samples at a lower VAF. This may be achieved by optimising cfDNA extraction methods and increasing plasma volume per patient. It is important to note that the aforementioned previous studies, focused solely on *EWSR1* fusion identification whereas this study investigated a wide range of sarcoma fusions. It is possible that different sarcoma entities or fusion types can affect the level of ctDNA shedding, or detectability by NGS capture methods. Additionally, our series included patients undergoing treatment or having received treatment shortly before cfDNA sample collection, which could have affected the fraction of ctDNA present in the samples.

The *ASPCR1-TFE3* fusion in the case of alveolar soft part cell sarcoma (M5), which was undetected in both tissue and ctDNA samples, had a unique average read depth of 1332× and 867×, respectively. Analysis of the original whole genome sequencing data for the tissue sample showed that the translocation breakpoint occurred at chromosomal positions chr17:82002602 and chrX:49036241 [24,25,26]. Unfortunately, the coverage in the sarcoma-specific NGS panel for these regions is incomplete, due to the presence of highly repetitive elements, and did not cover the specific breakpoints. No pathogenic SNV mutations were called from tumour tissue or cfDNA sample using this pan-sarcoma NGS panel that could be used to control for the presence of ctDNA. However, this is not an uncommon finding in fusion-positive sarcomas due to their low mutational burden.

The main limitation of this study is the relatively small cohort of patients and low amount of cfDNA available for analysis. The cfDNA material used was not collected purposefully for this study and was instead retrieved from biobank archives. The data presented therefore show the robustness of the assay, using samples in less than optimal conditions as opposed to freshly collected material with optimal yields. Additional samples with higher plasma volumes are required to fully validate the use of sarcoma-specific NGS panels for liquid biopsies. We performed a brief descriptive analysis and found no notable difference in structural variant detection from cfDNA based on tumour burden, treatment received or localisation of metastasis in these patients. As previously mentioned, it is possible that sarcoma subtype or fusion types are relevant factors in cfDNA testing, as is the effect of recent or ongoing systemic treatment. It is also possible that tumour size may impact the amount of ctDNA released and therefore, fusion detection. Shulman et al. have shown that 83.3% of Ewing’s sarcoma patients with a tumour size greater than 8 cm had detectable ctDNA, compared to 33.3% of patients with a tumour size less than 8 cm [22]. In order to test this hypothesis, a larger cohort of patients with different sarcoma entities or fusion types and varied tumour sizes would be required.

## 4. Materials and Methods

### 4.1. Ethics Approval and Consent to Participate

Tissue and plasma samples were used in accordance with the regulations of the tissue and liquid banks of National Center for Tumor Diseases (NCT) Heidelberg and after approval by the ethics committees of Heidelberg University (protocol number S-206/2011). Written informed consent was obtained prior to the study. Research was conducted in accordance with the Declaration of Helsinki.

### 4.2. Tumour Specimen Collection

A total of 24 DNA samples extracted from matched fresh frozen sarcoma tissue (*n* = 12) and 6–8 mL of plasma (*n* = 12) were obtained from the German Cancer Research Center, Heidelberg, Germany. DNA from tumour specimens was isolated using the AllPrep DNA/RNA/Protein Mini Kit (Qiagen, Hilden, Germany), followed by quality control and quantification using a Qubit 2.0 Fluorometer (Life Technologies, Carlsbad, CA, USA), a 2200 TapeStation system and a 2100 Bioanalyzer system (Agilent Technologies, Santa Clara, CA, USA). cfDNA from plasma samples was isolated using the QIAamp MinElute ccfDNA Midi Kit (Qiagen, Hilden, Germany). Quantification and quality control were done using a Qubit 3.0 Fluorometer (Life Technologies, Carlsbad, CA, USA) and a 2100 Bioanalyzer System (Agilent Technologies, Santa Clara, CA, USA).

#### Preparation of DNA Libraries from Matched Fresh Frozen Tumour Samples

DNA from the matched tissue tumour samples were prepared as previously described in McConnell et al. 2020, using the KAPA HyperPlus Kit (Roche Sequencing Solutions, Inc., Pleasanton, CA, USA), IDT dual index adapters (Integrated DNA Technologies, Inc., Coralville, IA, USA) and associated SeqCap target enrichment reagents (Roche Sequencing Solutions, Inc., Pleasanton, CA, USA) according to the manufacturer’s protocol, including dual size selection of the libraries (250–450 bp) [23]. Sequencing was performed using the NextSeq 500 Mid Output Kit v2.5 (150 Cycles) and analysis was performed using Illumina’s Basespace.

### 4.3. Preparation of Libraries from Sarcoma cfDNA Samples

Between 3 and 16.5 ng of DNA were used for library preparation using the KAPA HyperPlus kit (Roche Sequencing Solutions, Inc., Pleasanton, CA, USA) and IDT dual index adapters (Integrated DNA Technologies, Inc., Coralville, IA, USA). DNA was end-repaired, A-tailed and indexed adapters were ligated in accordance with the manufacturer’s protocol. Libraries were amplified using 8 PCR cycles before dual size selection. All 12 cfDNA sample libraries were pooled together in equal amounts. Libraries were hybridised overnight (20 h) using 1 μg of the pooled libraries and the sarcoma-specific custom designed biotinylated DNA baits (NimbleGen SeqCap EZ library, Roche NimbleGen, WI, USA). Sequencing was performed on a NovaSeq 6000 using the S1 Reagent Kit (200 cycles) (Illumina, San Diego, CA, USA) and standard workflow in accordance with the NovaSeq 6000 Sequencing System Guide (Illumina).

### 4.4. Sequencing and Structural Variant Data Analysis

Base calls and quality scores were provided by the instrument using real time analysis (RTA 2.0). For tissue samples, FASTQ data files were aligned to the hg38 build of the human reference genome using the Isaac Aligner (iSAAC-03.16.02.20). For cfDNA samples, FASTQ files were generated using the standalone bcl2fastq program (version 1.8.4) following demultiplexing and alignment. Translocations were detected from the aligned sequence data using Manta Structural Variant Caller (v0.28.0). Any samples with undetected translocations with the analysis pipeline were manually analysed using Integrative Genomics Viewer (IGV) by colour coding reads by insert size and grouping alignments by chromosome of mate [27,28]. Variant allele frequency was estimated for each structural variant by counting the number of alternative reads at the translocation breakpoint and dividing by the overall read depth at that chromosomal position.

### 4.5. Copy Number Variation Data Analysis

DNA copy number analysis was performed with CNVPanelizer R package Version 1.16.0, which is based on a subsampling strategy to predict copy number variation (CNV), to detect *MDM2* and *CDK4* amplification. CNVpanelizer compares *MDM2/CDK4* amplified samples with the non-amplified pool of fusion-positive samples [23,29].

## 5. Conclusions

This sarcoma-specific fusion gene panel has previously shown to be highly efficient in identifying translocations in sarcoma tissue samples and therefore clinically useful as a diagnostic method, using both resected tumour tissue and small biopsies [23]. This panel applied to plasma may provide benefits in aiding the diagnosis when a tissue sample is unavailable and monitoring disease using cfDNA as variants can be detected in at VAF < 1%. To our knowledge, this is the first sarcoma-specific targeted gene panel to identify multiple sarcoma translocations and gene amplifications in cfDNA samples. Five distinct sarcoma translocations and *MDM2* amplification were detected in this ctDNA series, with as little as 3.2 ng of cfDNA input. However, increased cfDNA yield and sequencing depth is required before it can be implemented in clinical practice.

## Figures and Tables

**Table 1:** Clinical information for patients.

Sample ID	Gender	Age at Diagnosis	Diagnosis	Structural Variant Detected	Date of Diagnosis (Month/Year)	Primary Tumour Location	Location of Metastases	Last Treatment Prior to Sampling	Time Elapsed between Last Treatment and cfDNA Sampling
M1	M	21	Alveolar Rhabdomyosarcoma	*PAX3-FOXO1*	11/2017	Hand	Intraabdominal, pleural sarcomatosis	Irinotecan	Ongoing
M2	M	65	Extraskeletal myxoid chondrosarcoma	*MEAF6-PHF1*	11/2018	Chest wall	Lung	Radiotherapy	3 months
M3	M	26	Ewing′s Sarcoma	*EWSR1-FLI1*	10/2016	Thigh	Lung, lymph nodes	Irinotecan, Temozolomide	Ongoing
M4	F	19	Undifferentiated round cell sarcoma	*CIC-DUX4*	11/2018	Abdomen	Lung, CNS	Vincristine, Actinomycin D, Cyclophosphamide	Ongoing
M5	F	40	Alveolar soft part cell Sarcoma	*ASPSCR1-TFE3*	04/2004	Thigh	Lung, heart, liver, abdomen, CNS	Pazopanib	Ongoing
M6	M	59	Dedifferentiated liposarcoma	*MDM2/CDK4* amp	05/2018	Abdomen	Liver, colon, lymph nodes, lung	Eribulin	Ongoing
M7	F	56	Synovial Sarcoma	*SS18-SSX2*	09/2017	Lung	Lung, pleural sarcomatosis, abdominal	Radiotherapy	1 year
M8	F	38	Alveolar Rhabdomyosarcoma	*PAX3-FOXO1*	07/2017	Abdomen	Abdominal	Gemcitabine, Docetaxel	One month
M9	M	65	Synovial Sarcoma	*SS18-SSX2*	03/2016	Lung	Pleural sarcomatosis, lymph nodes, bone	Trabectedin	3 weeks
M10	M	54	Myxoid Liposarcoma	*FUS-DDIT3*	01/07/2013	Retroperitoneum	N/A	Doxorubicin, Olaratumab, surgery	4 months
M11	M	54	Ewing′s Sarcoma	*EWSR1-FLI1*	10/05/2017	Thigh	Lung	Trabectedin	Ongoing
M12	M	56	Clear cell sarcoma	*EWSR1-ATF1*	16/03/2017	Thigh	Lung, Kidney, lymph nodes	Cisplatin, Etoposide, Ifosfamide	One month

Note: M: male, F: female, amp: amplification, N/A: not applicable, Ongoing: treatment ongoing at time of cell-free DNA (cfDNA) sampling.

**Table 2:** Sample sequencing metrics.

Metrics	Tumour Tissue		cfDNA	
	Average	Range	Average	Range
Total PF Reads	27,924,669	13,533,026–44,137,640	156,632,804	138,511,518–187,883,946
Unique PF Reads	12,220,362	3,814,200–15,097,928	12,677,104	10,214,908–15,493,964
Mean Target Coverage Depth	2390×	675×–4374×	1026×	842×–1270×
Percentage of Duplicates	57.63%	42.88–76.30%	93.62%	92.31–95.06%

Note: PF = passing filter.
